# The Lymphedematous Limb as a Donor Site for Breast Fat Grafting

**DOI:** 10.1097/GOX.0000000000005824

**Published:** 2024-05-17

**Authors:** Shahnur Ahmed, Ganesh Mohan, Luci Hulsman, Arin K. Greene, Muhammad Shaheen, Mithun Sinha, Aladdin H. Hassanein

**Affiliations:** From * Division of Plastic Surgery, Indiana University School of Medicine, Indianapolis, Ind.; † Department of Plastic and Oral Surgery, Boston Children’s Hospital, Harvard Medical School, Boston, Mass.; ‡ Department of Pathology, Indiana University School of Medicine, Indianapolis, Ind.

## Abstract

Breast cancer–related lymphedema results in chronic upper limb swelling with subcutaneous deposition of fluid and fibroadipose tissue. Morbidity includes psychosocial distress, infection, and difficulty using the extremity. Operative management includes excisional procedures such as suction-assisted lipectomy to reduce abnormal subcutaneous fibroadipose tissue to improve limb volume. Patients who have had postmastectomy breast reconstruction often benefit from fat grafting. This report introduces the concept of fat grafting the breast using the lymphedematous arm as a donor site. This technique improves the volume of the limb by removing the excess subcutaneous adipose, and at the same time reconstructs the breast without adding a donor site not related to the breast cancer–related lymphedema.

**Takeaways**

**Question**: Does using the lymphedematous limb for a donor site for breast fat grafting benefit breast cancer–related lymphedema (BCRL) patients undergoing breast reconstruction and concurrent lymphedema treatment?

**Findings**: All breast fat–grafted patients reported improvement in tightness of the affected arm and cosmetic appearance of the breast. There were no donor site complications in fat-grafted patients.

**Meaning**: Patients with BCRL who undergo liposuction may benefit from breast fat grafting. The lymphedematous arm can provide a large amount of donor adipose to the breast. This technique therapeutically treats BCRL and avoids creating another surgical site not related to the breast cancer.

## INTRODUCTION

Breast cancer–related lymphedema (BCRL) results in chronic limb enlargement from subcutaneous fluid, adipose deposition, and fibrosis.^1^ One-third of patients who undergo axillary lymph node dissection for locally advanced breast cancer or biopsy-proven metastatic disease develop lymphedema.^1^ Morbidity includes cellulitis, functional impairment, and decreased quality of life.^2^ Management of lymphedema includes compression, microsurgical physiological procedures, and excisional procedures such as liposuction.^3,4^ Patients with BCRL typically have had breast cancer managed with mastectomy or partial mastectomy and often undergo breast reconstruction. Fat grafting is an adjunct in breast reconstruction to fill contour deficiencies; 78,000 cases are performed annually in the United States.^5^ The purpose of this study was to introduce the concept utilizing the lymphedematous limb for a donor site for breast fat grafting in patients undergoing breast reconstruction and concurrent lymphedema treatment.

Following approval from the institutional review board, a single-center retrospective case series was performed for patients with BCRL who underwent liposuction as excisional treatment by the senior author (AHH). All patients underwent a preoperative lymphoscintigram to confirm lymphedema.^6^ Tumescence was applied, and patients underwent liposuction of the lymphedematous arm and forearm.^6^ Fat graft was procured from lipoaspirate using a lactated Ringer’s filtration system (Revolve, Allergan, Chicago, Ill.). [See Video (online), which displays fat grafting using lymphedema lymph as donor site. Suction-assisted lipectomy is performed on the lymphedema limb. After fat is harvested, fat grafting is performed for breast contouring.] Histological analysis of adipose obtained from the lymphedematous extremity was compared with control subaxillary adipose tissue from a normal, nonradiated limb using hematoxylin and eosin (H&E) and Masson’s trichome staining to evaluate fibroadipose changes related to lymphedema. Demographic information, body mass index (BMI), diabetes, smoking, length of lymphedema, lipoaspirate volume, limb circumference, and volume of fat graft were recorded. Outcome variables included subjective improvement of the arm and breast volume, as well as complications.

**Video 1:** displays fat grafting using lymphedema lymph as donor site. Suction-assisted lipectomy is performed on the lymphedema limb. After fat is harvested, fat grafting is performed for breast contouring

Thirteen patients underwent upper extremity liposuction of BCRL between 2019 and 2023. Four individuals (30.8%) had lipoaspirate from the treated lymphedematous limb used for breast fat grafting, and each patient had only one fat graft session. The average age was 50.3 ± 10.1 years, and mean BMI was 33.8 ± 5.2 kg/m^2^. All patients had a diagnosis of International Society of Lymphology stage II lymphedema. The average length of lymphedema diagnosis was 4.8 ± 3.8 years (range 2–10 years). The mean amount of lymphedema lipoaspirate was 1375 ± 467.9 mL (range 1000–2200 mL). The average amount of breast fat graft was 142.5 ± 130.7 mL (range 20–270 mL). Three patients had unilateral breast fat grafting to the ipsilateral breast, and one patient had fat grafting to the bilateral breasts. There were no donor site complications (pigment changes, incisional pain, surgical site infection) in fat-grafted patients. None of the fat-grafted patients had postoperative cellulitis. The proximal lymphedematous forearm was reduced by 16.1% (23 ± 2.5 cm compared with 19.3 ± 0.6 cm) after liposuction at 297 ± 93.3 days postoperatively. All fat-grafted patients reported improvement in tightness of the affected arm and appearance of the breast. The mean length of follow-up of breast fat–grafted patients who had improvement of their lymphedema was 249.5 days (range 150–342 days).

**Fig. 1.**
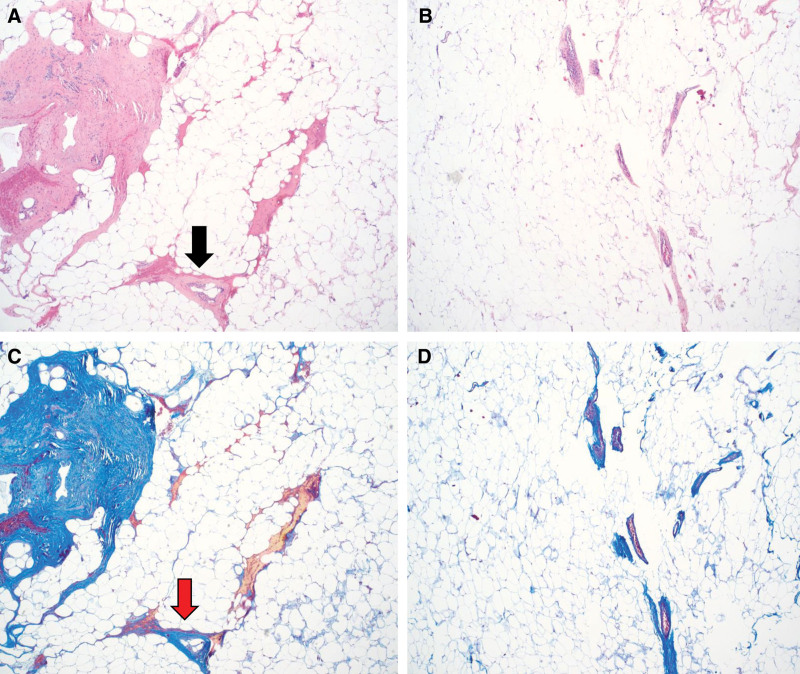
Histology of fat from lymphedematous limb compared with normal adipose. Hematoxylin and Eosin (H&E) of lymphedematous tissue (A) and normal adipose (B). Note fibroadipose tissue with mild to moderate perivascular fibrosis and mononuclear cell infiltrate (black arrow). Masson’s trichome staining of collagen to assess fibrosis in lymphedema adipose (C) and normal adipose (D). Control fibroadipose tissue stained with trichome (red arrow) shows minimal perivascular fibrous tissue without significant inflammation.

Histology exhibited mild to moderate perivascular fibrosis with mononuclear cell infiltrate in lymphedema tissue compared with control adipose tissue that showed minimal perivascular fibrous tissue without significant inflammation (Fig. 1).

## DISCUSSION

There is no cure for lymphedema. The limb enlarges from subcutaneous fluid and fibroadipose deposition.^7^ Suction-assisted lipectomy can reduce limb volume by up to 73% with removal of the abnormal subcutaneous fat.^8^ Liposuction improves quality of life and decreases the frequency of cellulitis.^8^

Fat grafting commonly is performed for breast reconstruction following the removal of cancerous breast tissue. It can improve rippling and superior pole deficiencies, particularly in prepectoral implant reconstruction; add volume and contour enhancement in autologous reconstruction; fill postlumpectomy defects; and be serially applied as the primary postmastectomy reconstructive modality.^9^ Frequent donor sites for breast fat grafting include the abdomen, thigh, or hip. Donor site morbidity from these sites include contour abnormalities, pigment changes, and chronic pain.^2^

Our report describes the use of the lymphedematous limb as a donor site for breast fat grafting. Advantages of this technique compared with other donor sites are as follows: (1) the volume of the lymphedematous extremity is improved, and (2) the upper limb can serve as a source of excess donor fat in patients with low BMI who may otherwise have limited donor sites. A previous report has described the use of the lymphedematous extremity as a donor site for aesthetic fat grafting to the face, and patients reported overall improvement of general appearance, skin texture, and self-esteem.^10^ They theorized that lymphedematous fat may be a superior filler graft compared with other sites based on high hyaluronic acid content compared with standard formulation of facial fillers.^10^ Although the adipose may be more fibrous, histology indicated mild perivascular fibrosis. Our initial impression is that fat graft resorption using lymphedematous adipose is similar to fat from other donor sites. Theoretically, however, the fibrosis component of lymphedematous adipose may behave more like a dermal graft with minimal to no resorption, which might reduce the overall resorption rate of the fat graft. This possibility can be a focus of further study. We use similar fat graft preparation and cannulas as other donor sites. Although long-term follow-up for fat graft retention was not evaluated in this study, magnetic resonance or three-dimensional imaging technology may further characterize fat graft retention. Further study is needed for histological assessment and long-term behavior of lymphedematous fat graft to the breast.

Our study was limited by a small sample who received fat graft to the breast. Although all patients reported subjective improvement of the arm and breast after liposuction and fat grafting, our study was limited because a validated metric of patient reported outcomes was not assessed.

## CONCLUSIONS

Patients with BCRL who undergo liposuction may benefit from breast fat grafting. The lymphedematous arm can provide a large amount of donor adipose to the breast. This technique therapeutically treats BCRL and avoids creating another surgical site not related to the breast cancer.

## DISCLOSURES

This work was supported by the National Institutes of Health grant K08HL167164 (to AHH). The content is solely the responsibility of the authors and does not necessarily represent the official views of the National Institutes of Health.
